# Naturally occurring canine laminopathy leading to a dilated and fibrosing cardiomyopathy in the Nova Scotia Duck Tolling Retriever

**DOI:** 10.1038/s41598-023-46601-2

**Published:** 2023-11-04

**Authors:** Danika L. Bannasch, Danielle T. Oertle, Julia Vo, Kevin L. Batcher, Joshua A. Stern, Joanna L. Kaplan, Ronald H. L. Li, Indiana E. Madden, Matthias Christen, Tosso Leeb, Nikhil Joshi

**Affiliations:** 1 Department of Population Health and Reproduction, School of Veterinary Medicine, University of California Davis, Davis, CA USA; 2 Department of Medicine and Epidemiology, School of Veterinary Medicine, University of California Davis, Davis, CA USA; 3 Department of Surgical and Radiological Sciences, School of Veterinary Medicine, University of California Davis, Davis, CA USA; 4 Institute of Genetics, Vetsuisse Faculty, University of Bern, 3001 Bern, Switzerland; 5 Bioinformatics Core, UC Davis Genome Center, University of California, Davis, CA USA

**Keywords:** Animal breeding, Clinical genetics, Consanguinity, Genetic association study, Genomics, Genotype, Inbreeding, Medical genetics, Genetics

## Abstract

Dilated cardiomyopathy (DCM) is characterized by decreased systolic function and dilation of one or both ventricles, often leading to heart failure or sudden death. Two 10-month-old sibling Nova Scotia Duck Tolling Retrievers (NSDTR) died acutely with evidence of dilated cardiomyopathy with myocardial fibrosis. Association analysis using two cases and 35 controls identified three candidate regions homozygous in the two cases. Whole genome sequencing identified a frameshift deletion in the *LMNA* gene (NC_049228.1:g.41688530del, NP_001274080:p.(Asp576ThrfsTer124)). Three retrospectively identified NSDTRs with sudden death before 2 years of age and severe myocardial fibrosis were also homozygous for the deletion. One 5 year old with sudden death and myocardial fibrosis was heterozygous for the deletion. This variant was not identified in 722 dogs of other breeds, nor was it identified to be homozygous in 784 NSDTR. *LMNA* codes for lamin A/C proteins, which are type V intermediate filaments that provide structural support to the nuclear membrane. In humans, *LMNA* variants can cause DCM with sudden death as well as diseases of striated muscles, lipodystrophy, neuropathies, and accelerated aging disorders. This frameshift deletion is predicted to affect processing of prelamin A into lamin A. Pedigree analysis in the NSDTR and functional evaluation of heterozygotes is consistent with a predominantly recessive mode of inheritance and possibly low penetrance in heterozygotes in contrast to people, where most pathogenic *LMNA* variants are dominantly inherited.

## Introduction

Cardiovascular disease is the fourth most common cause of death in dogs^1^ and one of the most common causes of death in humans^2^. Dilated cardiomyopathy (DCM) is the second most prevalent form of heart disease in dogs, accounting for 10% of cardiac diagnoses^3^. Characteristic findings of adult onset DCM in dogs include progressive dilation of the left or both ventricles with concurrent systolic dysfunction and eventual development of congestive heart failure or sudden death^4^.

While a number of phenocopies for DCM in dogs are reported, such as nutritionally mediated^5,6^, infectious disease-associated^7,8^, or metabolically mediated^9^, idiopathic canine DCM is considered a heritable disease^10^. Large breed dogs are more susceptible to adult onset DCM and several breeds, such as the Doberman Pinscher, Great Dane, Newfoundland, Boxer, Scottish Deerhound, and Irish Wolfhound, are over-represented. Mendelian early onset DCM has been identified in a number of breeds. A highly penetrant dominant missense variant in phospholamban was identified in a family of Welsh Springer Spaniels with young adult onset of DCM and sudden death^11^. Another highly penetrant recessive variant in the *RBM20* gene was identified in the Standard and Giant Schnauzer breeds, leading to significantly reduced lifespan^12^. A juvenile form of DCM has also been documented in the Portuguese water dog and Toy Manchester Terrier, although a causative variant has not been identified^13,14^. One challenge in identifying inherited forms of DCM leading to sudden death is that necropsies are not routinely performed.

Many different forms of DCM have been identified in humans. Nineteen genes with high evidence for causation come from different ontologies, underscoring the genetic complexity of the disease process^15^. One of the genes with strong evidence for causation is the *LMNA* gene which codes for lamin A and lamin C, two alternatively spliced isoforms that are type V intermediate filament proteins localized to the nuclear lamina^16^. Autosomal dominant dilated cardiomyopathy and conduction system disease may result from variants in *LMNA*^17^. Patients with *LMNA* variants are at risk for sudden death and end-stage heart failure^18^.

Over 450 disease causing variants in the *LMNA* gene lead to metabolic, nervous system, and skeletal and cardiac muscle diseases (UMD-LMNA at http://www.umd.be)^19,20^. The genotype–phenotype correlation is unclear and the same variants may be found in patients with distinct disease phenotypes^19,20^. The nuclear lamina has important roles in nuclear shape and structure, as well as functional roles in transcriptional regulation and heterochromatin organization. Disruption of these processes contributes to the diverse pathogenesis of laminopathies^21^.

A 10-month-old Nova Scotia Duck Tolling Retriever (NSDTR) was identified with a ventricular tachyarrhythmia and DCM after presentation for evaluation of gastrointestinal disease. Within 2 weeks of presentation the dog died suddenly. Phenotypic evaluation of family members, GWAS and whole genome sequencing identified a frame shift variant in the *LMNA* gene segregating with DCM and/or sudden death and myocardial fibrosis.

## Methods

### Phenotype

The proband dog had an echocardiogram and thoracic radiographs. The dog died suddenly and had a complete necropsy. An affected littermate had an echocardiogram and thoracic radiographs. The sire, dam and grand dam of these dogs as well as two siblings each had an echocardiogram. Normal echocardiographic values were determined using previously published reference ranges^22,23^. An echocardiographic diagnosis of DCM was made based on the presence of both reduced left ventricle (LV) systolic function (%FS < 25) and systolic LV enlargement. Pedigree analysis was performed by using an online database for the NSDTR (https://www.k9data.com/). Life span for NSDTR was also determined when available from this online database. A second related litter with three siblings with sudden death at 15 months of age was born 11 years previously. Necropsy findings on one of these dogs were available for review. An additional affected dog (not closely related to the first two litters) was identified by screening the Bannasch Laboratory DNA Database for any cases of sudden death within this breed. A full necropsy report was available for this dog. Upon release of a genetic test for dog breeders, two additional affected dogs that had died 2 years prior were identified and had undergone necropsy. DNA was extracted from formalin-fixed paraffin-embedded (FFPE) tissues to determine the genotypes of these two dogs (Supplementary Table S1). A full sibling to the sire of the proband had sudden death at 5 years of age and a complete necropsy was performed. Comparison of lifespan between dogs heterozygous for the *LMNA* variant and wildtype dogs was performed using an unpaired *t* test as implemented in Graphpad Prism v0.97 (Dotmatics, Boston, MA, USA).

### Samples used for genetic analyses

Three hundred and sixty-seven North American NSDTR DNA samples were used for this study. Thirty-five related NSDTR were used as controls for GWAS with two full sibling cases. One case, one obligate carrier and grandam of the proband were used for whole genome sequencing and variant calling. 300 NSDTR unrelated within 2 generations were genotyped for the *LMNA* variant identified. Additional related NSDTR were also genotyped to confirm the mode of inheritance (N = 32). An online database (https://www.k9data.com/) was used to identify related individuals. DNA samples were collected under Institutional Animal Care and Use Committees (IACUC) #s15356, 16892, 18561 and 22035. Four hundred and twenty-two DNA samples from NSDTR of mostly Swiss origin (351 Swiss, 57 other European countries, 14 unknown) from the Vetsuisse Biobank were used to estimate the allele frequency in Europe. None of those dogs had known reports of cardiac phenotypes. Blood samples were collected with the approval of the Cantonal Committee for Animal Experiments (Canton of Bern; permit BE 71/19). All sample collections were performed in accordance with the guidelines of the University of California Davis and the Canton of Bern respectively. The authors have complied with the ARRIVE guidelines in the design and execution of this project.

### DNA extraction

Genomic DNA was extracted from buccal swabs and EDTA blood samples using the Gentra Puregene Kit (Qiagen, Valencia, CA, USA). FFPE kidney or liver scrolls were extracted using Zymo Research’s (Orange, CA, USA) Quick-DNA FFPE Miniprep kit. For European samples DNA was extracted from EDTA blood samples according to standard methods using the Maxwell RSX Whole Blood DNA kit in combination with the Maxwell RSC instrument (Promega, Dübendorf, Switzerland).

### Genome-wide association study

The Illumina Canine HD BeadChip was used to generate genotype calls for 37 NSDTR, including two cases and 35 related NSDTR controls. PLINK v1.9 software was used to prune the data for minor allele frequency below 5% and individuals and single nucleotide variants (SNVs) with more than 10% missing genotypes^24^. A kinship matrix was implemented using GEMMA to correct for population stratification and a modified form of homozygosity mapping was performed. Only variants homozygous in the two cases were evaluated further 3636 variants were homozygous in the two cases. Bonferroni threshold was calculated for the 3636 variants at *P* = 0.05 and only SNV with significant associations were considered. A Manhattan plot was generated for SNV that were homozygous in the cases using the qqman package in RStudio^25^. UU_Cfam_GSD_1.0 dog reference genome assembly coordinates are used.

### Whole genome sequencing and variant calling

One case, one obligate carrier and the grandam of the case were whole genome sequenced. PCR free Library preparation and 2 × 150 bp Illumina paired-end sequencing was performed at the UC Davis DNA Technologies Core. The raw read data was filtered using HTStream (version 1.3.3)^26^ which included screening for contaminants (such as PhiX), overlapping reads, quality-based trimming, and adapter trimming. BWA MEM (version 0.7.17)^27^ and Samtools (version 1.14)^28^ were used to align the processed data to the canine genome ((UU_Cfam_GSD_1.0). Sequencing duplicates were marked using Picard tools (version 2.26.11) (broadinstitute.github.io/picard/). Variant calling was accomplished using the GATK (version 4.2.5.0)^29^ pipeline which included the following steps: 1. HaplotypeCaller to call variants in the gVCF format, 2. GenomicsDB to create a datastore to store the variant call data, 3. GenotypeGVCFs to perform joint genotyping on all samples. Finally, SnpEff (version 5.1)^30^ was used to add effect prediction to the final VCFs using GSD_1.0 annotation^31^. Whole genome sequencing data from an affected NSDTR, one obligate carrier and one likely carrier, and 128 unaffected control dogs (Bioprojects PRJNA776905, PRJNA377155 and PRJNA961733) were analyzed for protein-coding variants unique to the affected and carrier dogs. WebGQT^32^ was utilized to filter based on a recessive pedigree model for variants.

### Gene analysis

UU_Cfam_GSD_1.0 dog reference genome assembly was used to investigate the *LMNA* gene. IGV was used to visually confirm the variant^33^. NCBI RefSeq accession numbers NM_001287151.1 (mRNA) and NP_001274080.1 (protein) corresponding to the canine *LMNA* gene were used.

### Database searches and functional predictions

The Ensembl Variant Effect Predictor release 107 was used to predict the biological outcome of the identified candidate variant. Plink v1.9 software was used to evaluate the *LMNA* genotype from a VCF file comprising 722 canine genomess^34^.

### PCR and Sanger sequencing

PCR primers 5′-GAAGAGCCAGAGGAGATGGA and 5′-GTGCGAGCAGGAGTACAGAG were used to generate an amplicon containing the *LMNA* variant NM_001287151.1:c.1726del. PCR products from North American samples were directly Sanger sequenced on an Applied Biosystems 3500 Genetic Analyzer after enzymatic cleanup. Sanger sequences were analyzed using SnapGene^®^ software (Dotmatics, Boston, MA, USA). European samples were sequenced on an ABI 3730 DNA Analyzer (Thermo Fisher Scientific, Reinach, Switzerland) and viewed in Sequencher 5.1 software (Gene Codes, Ann Arbor, MI, USA).

### Quantification of collagen in cardiac muscle

FFPE blocks from dogs with no known cardiac conditions euthanized between 17 months and 3.5 years of age were used as references to examine fibrosis levels in diseased heart tissue. Masson’s trichrome stained slides of left ventricle from three control (one German shepherd, one Labrador retriever and one Rottweiler mix) and one homozygous case were prepared by the Anatomic Pathology Service at the UC Davis Veterinary Medical Teaching Hospital. Slides from a second and third homozygous case were prepared by Pathobiology at University of Guelph’s Ontario Veterinary College and the Veterinary Diagnostic Laboratory at the University of Arkansas respectively. Ten randomly selected fields of cross-sectional myocardium were imaged from each slide at 20 × magnification on a Revolve hybrid digital microscope. Fibrosis, quantified by collagen tissue proportion, was measured using Masson Trichrome vectors in Fiji’s Colour Deconvolution 2 plug-in^35,36^. Blue-stained collagen pixels were quantified in deconvolution Colour_1 according to Yen’s thresholding method and divided by total tissue pixels counted according to Intermodes thresholding^37,38^. Normality was tested using the Anderson–Darling test and an unpaired two-tailed *t* test was used to compare the collagen-to-total tissue proportion in *LMNA* mutant dogs to wildtype controls. Statistical tests and graphs were prepared using Prism Version 9.2.0

### Verification of the mutant RNA

RNA was extracted from the white blood cells of a carrier and reverse transcribed to cDNA to verify the sequence of the mutant transcript. The C-terminal domain of *LMNA* was amplified with primers 5′-GAGGACGATGAGGATGAGGA (Exon 10) and 5′-AGCAGGGGAGATTGACATAGA (3′UTR). RT-PCR products were TOPO TA cloned and plasmids purified by column miniprep. Inserts were sequenced by the UCDNA Sequencing Facility at UC Davis.

## Results

A 10-month old male NSDTR presented for diarrhea and vomiting. On physical exam an abnormal rhythm was auscultated and the dog was referred to a cardiologist. Paroxysmal ventricular tachycardia, severe DCM and mild suspected mitral valve dysplasia were diagnosed. Two weeks later the dog died suddenly. Concomitantly, a female littermate was reported to be experiencing inappetence and lethargy. The littermate was also diagnosed with DCM and sinus tachycardia with a left bundle branch block and died 2 weeks later. The parents, paternal granddam and two apparently unaffected siblings had echocardiograms and showed no evidence of DCM or heart disease at the time of screening (Table 1). Both affected puppies showed significant reduction in fractional shortening (< 25%) along with systolic and diastolic LV and LA dilation consistent with a diagnosis of advanced DCM^22,23^.

**Table 1:** Cardiac findings in proband and relatives (Litter A).

	Sex	Weight (kg)	LVIDd (cm)	LVIDs (cm)	LVIDDn	Fractional shortening (%)	LA:Ao
Proband	M	15.4	4.5	3.6	2	20	2.4
Affected sibling	F	11.3	3.9	3.4	1.9	13.5	2.5
Unaffected sibling	M	17.6	3.6	1.7	1.6	53	1.3
Unaffected sibling	M	20.1	3.7	2.2	1.5	39	1.2
Sire	M	19.5	2.8	1.9	1.2	35	1
Dam	F	16.3	3.3	1.6	1.4	54	1.1
Granddam	F	19.4	3.3	2.4	1.4	27.3	1.3

*LVIDd* left ventricle internal diameter diastolic, *LVIDs* left ventricle internal diameter systolic, *LVIDDn* left ventricle size normalized for weight, *LA:Ao* left atrial to aortic root ratio.

**Figure 1:**
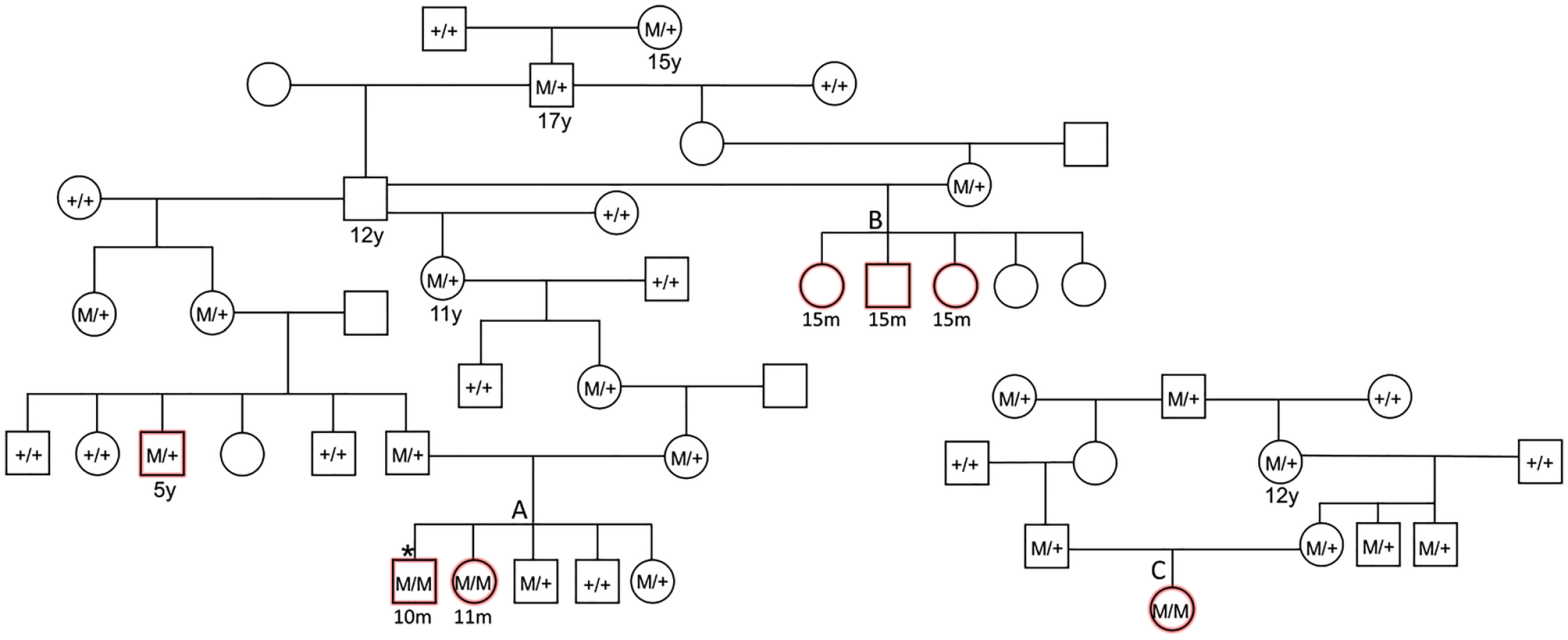
Pedigree of affected dogs. Affected dogs are outlined in red. Squares represent male dogs and circles represent female dogs. The asterisk indicates the proband case. Numbers below the symbols indicate lifespan of dogs when available. Genotype data for the *LMNA* variant is shown inside each symbol for the dogs with DNA available. A M indicates the mutant allele (NC_049228.1:g.41688530del) and a + indicates the normal allele. The three litters that produced affected puppies are indicated with letters A, B and C. The three litters have inbreeding loops. Age at death is noted under the pedigree symbols in months (m) or years (y).

Pedigree analysis of the litter revealed that there was a recent inbreeding loop (Litter A, Fig. 1). A litter related to the proband had three puppies that died suddenly at ~ 15 months of age within a few weeks of one another (Litter B, Fig. 1). The proband and one puppy from the second litter had necropsies performed and both showed evidence of significant myocardial fibrosis. DNA samples were not available from the affected dogs from the second litter (Litter B, (Supplementary Table S1 and Fig. 1).

**Figure 2:**
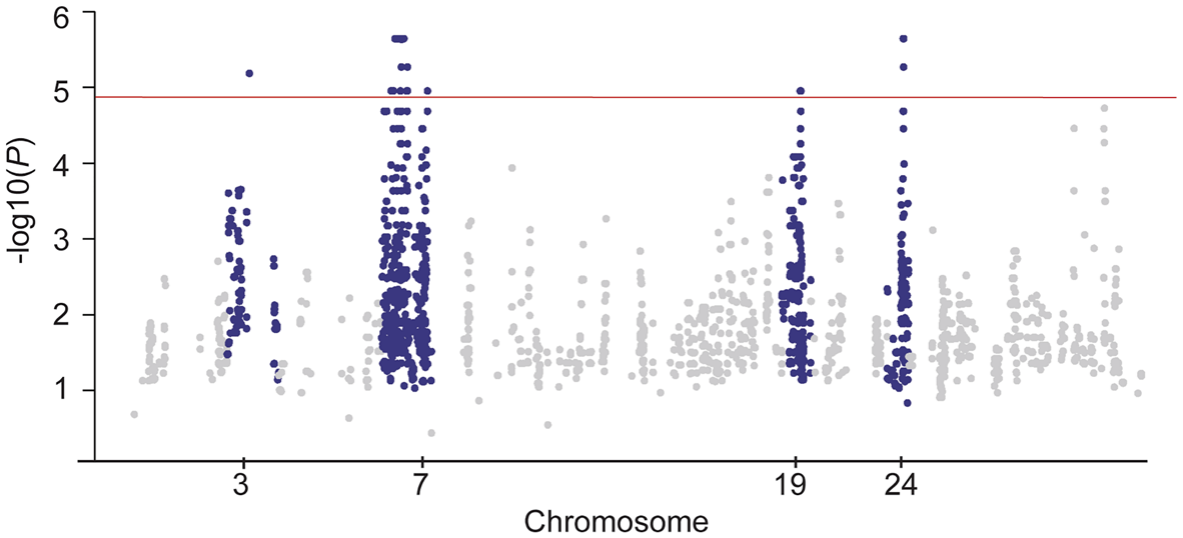
Manhattan plot. The − log^10^*P* value for SNV that were homozygous in two affected littermates are plotted by chromosome. Four suggestive regions were identified on CHR 3, 7, 19 and 24 based on Bonferroni significance (1.4 × 10^−5^) shown by the red line.

Although DNA was initially only available from two cases, a genome-wide association study was performed using the two cases and 35 related, clinically unaffected NSDTR. Four candidate chromosomal regions homozygous in the cases were identified: chr7 (18693546–74786398), chr19 (33841298–34756758), chr24 (40956242–41731762), and a single SNP on chr3 (54049478)(Fig. 2).

### Identification of candidate causative variants

Whole genome sequence was obtained from the affected sibling of the proband, the sire and paternal granddam. Candidate causal variants for DCM were identified by applying a hard filtering strategy on protein coding variants. The affected dog was required to be homozygous for the non-reference allele, the two presumed carriers were required to be heterozygous, and 128 previously sequenced dogs from other breeds were required to be homozygous for the reference allele.

Using this strategy two variants were identified. One was a 9 base pair deletion in the 5′-UTR of *POLI* (NC_049222.1:g.21302953-21302961del) on CFA1 (UU_Cfam_GSD_1.0). This variant did not fall within our candidate regions. The second variant was a single base deletion in the *LMNA* gene (NC_049228.1:g.41688530del) (UU_Cfam_GSD_1.0). *LMNA* is located on CFA 7 within the largest region of homozygosity identified in the association analysis. Based on its function and location, the *LMNA* variant was a very likely candidate causal variant. The *LMNA* variant causes a frameshift mutation, NM_001287151.1:c.1726del, NP_001274080:p.(Asp576ThrfsTer124).

**Figure 3:**
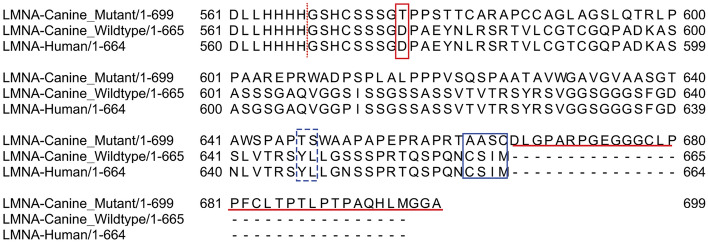
LMNA protein alignment of mutant dog, wildtype dog and human. The deletion results in a frameshift starting at codon 576 (red box). The predicted mutant protein contains 34 amino acids beyond the normal carboxy-terminus (red underline). The *CaaX* motif, CSIM, (blue box) is required for initial processing of prelamin to mature lamin A. The last 13 amino acids of prelamin are cleaved between a tyrosine and leucine residue (blue dotted box) to complete the processing of mature lamin A. Lamin C is cleaved between amino acids 566 and 567 (red dashed line) for processing and is predicted to occur normally.

In order to verify splicing of exons flanking this insertion, Sanger sequencing of RT-PCR products generated from RNA isolated from white blood cells from a heterozygous dog confirmed the intron–exon boundaries in the mutant and wildtype alleles for exons 10, 11 (which contains the deletion) and 12. The frameshift does not introduce a premature stop codon and is predicted to result in a mutant protein with 124 altered amino acids at its C-terminus (34 amino acids longer than the wildtype protein). Cleavage of lamin C is not predicted to be affected since residues 567 and 568 are unaltered. Due to the altered C-terminus, prelamin A processing is predicted to be disrupted. The first step of processing is farnesylation of the carboxy-terminal CaaX motif which is no longer present in the predicted mutant protein. This step is usually followed by cleavage at AA 646 which is also altered (Fig. 3). Expression from the mutant allele is predicted to lead to normal lamin C, aberrant C-terminal non-farnesylated prelamin A, and absent mature lamin A.

Genotyping for the *LMNA* variant in the pedigree of the proband demonstrated that the variant segregated with disease and was consistent with a recessive mode of inheritance (Fig. 1). An additional dog with sudden death at 12 months of age and necropsy findings of severe myocardial fibrosis was identified in our collection of DNA samples from this breed. It was confirmed to be homozygous for the *LMNA* variant. Genotypes from relatives of this dog were consistent with a recessive mode of inheritance (Litter C, Fig. 1). Four dogs heterozygous for the *LMNA* variant were evaluated by echocardiography and did not show evidence of cardiac disease (Table 1 and Supplementary Table S1). One 8-year-old heterozygote also had a 24-h portable electrocardiogram monitor that showed no evidence of arrhythmias (Supplementary Table S1). Five dogs in the pedigree that were heterozygous or obligate carriers lived from 11 to 17 years. A 5-year old heterozygote sibling to the sire of Litter A had sudden death and a necropsy which showed myocardial fibrosis.

We genotyped 300 additional NSDTR from North America unrelated to 2 generations to the proband; none of the dogs were homozygous for the *LMNA* variant and the carrier frequency was 8.7%. The carrier frequency in an additional 422 NSDTRs from a European cohort was 0.2%. None of the dogs were homozygous for the variant. The *LMNA* variant also was not present in 722 whole genome sequenced dogs from various sources in the public domain^34^. Evaluation of the age of death of heterozygotes (N = 20) compared to wildtype (N = 44) dogs did not reveal a statistically significant difference in age (Het—mean age 12.12 years, WT—mean age 13.12 years).

**Figure 4:**
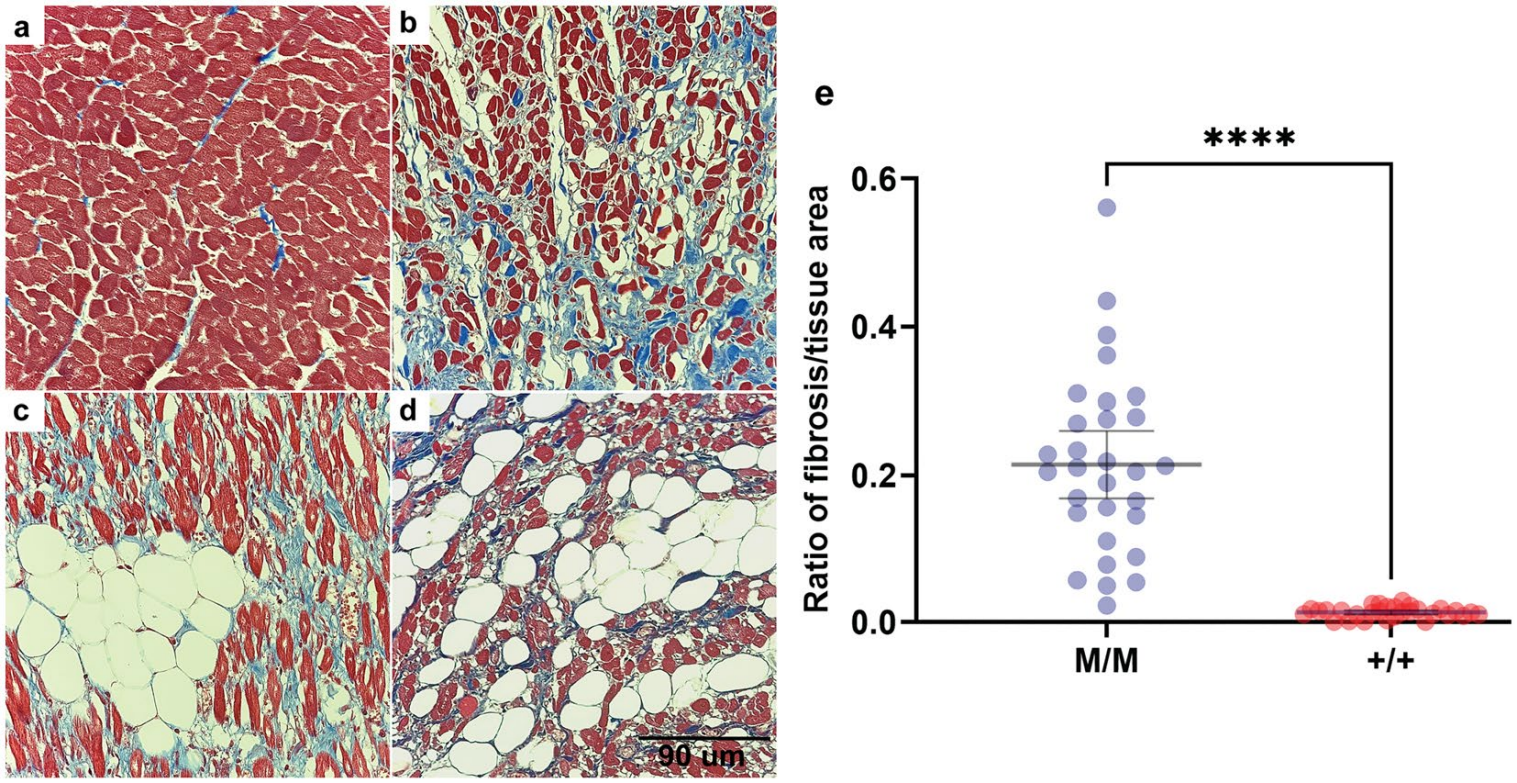
Severe myocardial Fibrosis. Sections are stained with Masson’s trichrome. Panel (**a**) is from a normal dog for comparison. Panels (**b**–**d**) are from three cases homozygous for the *LMNA* variant who died suddenly. Panel (**e**) showed the quantitative difference of the ratio of fibrosis/tissue between cases (M/M) and controls (+/+). The black calibration bar in panel (**d**) is 90 µm in length and the P value was < 0.0001.

After the NSDTR breeder community was informed about the presence of a genetic variant that could cause sudden death, two additional dogs were identified that died suddenly at a young age and had necropsies performed. Genotyping these dogs from FFPE samples demonstrated that both were homozygous for the *LMNA* variant (Supplementary Table S1). In order to quantify myocardial fibrosis, Masson’s trichrome stained left ventricle for these two cases and the proband were compared to three controls (Fig. 4a–d). Significant differences in blue staining consistent with cardiomyocyte fibrosis was observed between cases and controls (Fig. 4e). Cases were also observed to have a variable but subjective increase of adipocytes infiltrating the myocardial sections.

## Discussion

Young NSDTRs were identified that died suddenly and were diagnosed with DCM prior to death or severe myocardial fibrosis upon necropsy after sudden cardiac death. Genome-wide association analyses and whole genome sequencing identified a deletion resulting in a frameshift in the *LMNA* gene. Five young affected individuals were homozygous and one older affected dog was heterozygous for the deletion. Four heterozygotes were phenotypically normal and did not show abnormalities by echocardiography or ambulatory electrocardiogram when available. The frameshift variant identified in *LMNA* is predicted to result in normal production of lamin C. However, any expressed prelamin A would have a modified C-terminal tail, most likely interfering with the processing into mature lamin A due to disruption of key residues required for post-translational processing.

Nuclear lamins are an integral part of the inner nuclear membrane where they form an interlinked protein lattice structure that provides nuclear structural integrity and acts as a scaffold for chromatin organization^39,40^. Variants in *LMNA* result in more than 15 distinct tissue-specific diseases and over 450 disease causing variants have been identified in this gene in humans^19,20^. Dilated cardiomyopathy and conduction system disturbances are a common sequela of *LMNA* variants and can occur in combination with other phenotypes or in isolation^17,41–46^. In humans, 6–33% of cases presenting with both DCM and cardiac conduction defects are due to variants in *LMNA*^41,47^. *LMNA*-related DCM are inherited in an autosomal dominant manner either due to missense variants or truncating frameshift variants which are predicted to alter both lamin A and lamin C proteins^41,47^.

The *LMNA* variant identified in NSDTR is predicted to produce exclusively non-farnesylated prelamin A and normal lamin C. Mice that produce only non-farnesylated prelamin A develop DCM with mild to moderate fibrosis and die prematurely; however, in this mouse model no lamin C is produced^48^. Mice without lamin A or prelamin A have also been shown to be phenotypically normal^49^. The accumulation of non-farnesylated prelamin A is a gain of function and would be predicted to result in a dominant phenotype, however in the NSDTR, there is evidence for incomplete dominance with low penetrance in heterozygotes. The severe phenotype in homozygous mutant NDSTRs suggests loss of function of mature lamin A as a major contributor to the disease phenotype.

Similar to the mouse mutants which accumulate non-farnesylated prelamin A, affected NSDTR cardiac tissue showed significant fibrosis. Pathological myocardial fibrosis occurs at the first signs of conduction disturbances and prior to echocardiographic changes in humans with dominant variants in *LMNA*^50^. Cardiovascular magnetic resonance (CMR), a technique not used in veterinary medicine, has demonstrated 88% of asymptomatic or mildly symptomatic carriers of *LMNA* variants have myocardial fibrosis^51,52^. Recently, activation of the DNA damage response (DDR) pathway has been implicated in the induction of inflammation in laminopathies^53,54^. In fact, blocking the DDR pathway by disrupting the *Mb21d1* gene lead to increased survival and decreased apoptosis and fibrosis in *Lmna*-deficient mice^55^.

A human variant very similar to the one identified in the NSDTR is LMNA p.Thr655AsnTer49, which leads to an altered and extended C-terminus, although the frameshift occurs 80 codons later than in the NSDTR variant. This variant causes accumulation of non-farnesylated prelamin A^56^. People heterozygous and homozygous for this variant have lipodystrophy. Homozygotes can also have a cardiac phenotype characterized by DCM and/or rhythm/conduction disturbances and sometimes sudden death^57,58^. Interestingly there is no evidence for lipodystrophy in the NSDTR. One significant disease manifestation of lipodystrophy in humans is an increased risk of type 2 diabetes. Unlike humans, insulin resistant diabetes is not recognized clinically in dogs since their beta cells do not appear to be sensitive to hyperglycemia^59^. Even when obesity is induced in dogs, type 2 diabetes only develops with the addition of streptozotocin^60^.

One distinct difference between human cardiac laminopathies and the NSDTR and mouse mutants is the mode of inheritance. Virtually all human variants that lead to DCM and cardiac fibrosis are dominantly inherited with the exception of LMNA:p.Thr655AsnTer49 where some homozygotes showed cardiac defects^61^. Mouse *Lmna* mutants (both null and missense) also show a recessive mode of inheritance^48,62,63^. Four dogs that were carriers of the LMNA p.Asp576ThrTer124 variant received echocardiograms which showed no evidence of DCM (Table 1, Supplementary Table S1). In addition, other heterozygotes in this pedigree lived normal lifespans (N = 4; 11, 12, 15 and 17 years). However, the identification of a heterozygote with cardiac fibrosis and sudden death at 5 years of age suggests that the mode of inheritance might not be a simple recessive. On the other hand, It is possible that there are other risk factors that contributed to this dog’s death.

The clinical course of the NSDTR homozygous for the *LMNA* variant was similar in all six affected dogs. They were all young (10–15 months) and died suddenly. The proband was diagnosed with heart disease prior to sudden death but his history and clinical signs were gastrointestinal in nature. His littermate was subsequently diagnosed with DCM. The other affected homozygotes and the single heterozygote had no history of illness prior to sudden death. There is no evidence of incomplete penetrance when having two copies of the *LMNA* variant, however only a small number of homozygous dogs have been identified. In the two litters with multiple affected dogs, the age of onset was slightly different (Litter A 10 months and Litter B 15 months) opening the possibility of environmental or genetic modification of the phenotype. Importantly five cases were identified because their owners opted to have a complete necropsy performed. The importance of a necropsy for any young animal that dies suddenly cannot be overemphasized.

Familial DCM has not been described in the NSDTR dog breed prior to this study. Genotyping of NSDTR dogs from North America revealed a carrier frequency of 8.7% whereas European origin NSDTR dogs had a carrier frequency of 0.2%. By increasing awareness of this genetic defect within the NSDTR community we hope breeders are able to identify carriers and avoid pairings that would produce affected puppies.

In purebred dogs there are long regions of linkage disequilibrium which can challenge the identification of the causative variant underlying a phenotype^64^. The evidence that NM_001287151.1:c.1726del is a pathogenic variant is very strong^65^. The variant segregates with disease in distantly related families of NSDTR. The variant is not found in other breeds and was not found to be homozygous in 784 unaffected NSDTR. There is also a large body of evidence from human and mouse studies that *LMNA* variants can cause myocardial fibrosis and conduction system disturbances similar to what was seen in the NSDTR. Lastly the nature of the change to the LMNA isoforms is very similar but more severe than one seen in humans which can cause myocardial disease. The identification of LMNA p.(Asp576ThrfsTer124) as a pathogenic variant associated with sudden death and severe myocardial fibrosis provides a large animal model of a cardiac laminopathy. The underlying severe myocardial fibrosis seen in these young dogs also underscores the importance of inflammation and fibrosis to conduction disturbances of *LMNA* variants.

### Supplementary Information

Supplementary Table S1.

## Data Availability

The whole genome sequence data generated during the current study are available in the SRA repository, https://www.ncbi.nlm.nih.gov/sra, Bioproject PRJNA961733.
